# Flicker-Driven Responses in Visual Cortex Change during Matched-Frequency Transcranial Alternating Current Stimulation

**DOI:** 10.3389/fnhum.2016.00184

**Published:** 2016-04-26

**Authors:** Philipp Ruhnau, Christian Keitel, Chrysa Lithari, Nathan Weisz, Toralf Neuling

**Affiliations:** ^1^Centre for Cognitive Neuroscience, University of SalzburgSalzburg, Austria; ^2^Center for Mind/Brain Science, University of TrentoMattarello, Italy; ^3^Centre for Cognitive Neuroimaging, Institute of Neuroscience and Psychology, University of GlasgowGlasgow, UK

**Keywords:** alpha rhythm, brain oscillation, entrainment, frequency tagging, MEG, NIBS, steady-state response, tACS

## Abstract

We tested a novel combination of two neuro-stimulation techniques, transcranial alternating current stimulation (tACS) and frequency tagging, that promises powerful paradigms to study the causal role of rhythmic brain activity in perception and cognition. Participants viewed a stimulus flickering at 7 or 11 Hz that elicited periodic brain activity, termed steady-state responses (SSRs), at the same temporal frequency and its higher order harmonics. Further, they received simultaneous tACS at 7 or 11 Hz that either matched or differed from the flicker frequency. Sham tACS served as a control condition. Recent advances in reconstructing cortical sources of oscillatory activity allowed us to measure SSRs during concurrent tACS, which is known to impose strong artifacts in magnetoencephalographic (MEG) recordings. For the first time, we were thus able to demonstrate immediate effects of tACS on SSR-indexed early visual processing. Our data suggest that tACS effects are largely frequency-specific and reveal a characteristic pattern of differential influences on the harmonic constituents of SSRs.

## Introduction

Neural rhythms are prime candidates for a universal means of communication within and across brain regions and may code information from bits up to full objects (Engel et al., 2001; Buzsáki and Draguhn, 2004). A number of recent studies have thus attempted to entrain brain rhythms with external pacemakers by means of non-invasive brain stimulation (NIBS). An NIBS method widely applied in current cognitive neuroscience is transcranial alternating current stimulation (tACS; Thut et al., 2011; Antal and Paulus, 2013; Herrmann et al., 2013). Compared to classic electrophysiological research, tACS is in principle a more direct means to probe the role of brain oscillations in cognition: a strictly periodically alternating current is applied to modify brain rhythms directly that have been previously implicated with cognitive function. This way, different parameters of brain oscillations (e.g., amplitude, phase, frequency) become the independent variable and behavioral measures the dependent variable, which in turn allows for causal interpretations. Oscillations of various frequencies have been found to show tACS after-effects that appear brain state dependent. For instance, alpha band power (~10 Hz) was increased after 10 min of individual alpha frequency (IAF) stimulation (Zaehle et al., 2010), an effect lasting up to 30 min after stimulation (Neuling et al., 2013). tACS targeting different frequency bands and brain functions has also been shown to influence behavioral performance. As an example, stimulation within the theta frequency band (3–8 Hz) affects working memory performance (Polanía et al., 2012; Vosskuhl et al., 2015). Alpha tACS phase influences detection of near threshold stimuli in a phasic manner (Neuling et al., 2012a), while the IAF can be modulated by tACS, which in turn affects the multisensory double flash illusion (Cecere et al., 2015).

Although event-related activity and modulations in other frequencies has been successfully demonstrated during tACS using electroencephalography (EEG), attempts at investigating intrinsic brain oscillations at the stimulation frequency have proven to be challenging (Helfrich et al., 2014a,b; Voss et al., 2014). The main reason for this limitation is a heavy electrical artifact introduced by tACS that disallows analyses of spectral components of EEG/magnetoencephalographic (MEG) time series that are close to the stimulation frequency.

Recently, however, Soekadar et al. (2013) demonstrated that artifacts introduced by another NIBS method, transcranial direct current stimulation (tDCS), can be effectively suppressed by means of a beamformer source reconstruction of MEG sensor data. We successfully extended their approach to reconstruct brain activity during alpha-band tACS (Neuling et al., 2015). In that study we were able to demonstrate that two classes of mass neural activity, the parieto-occipital alpha rhythm and event-related responses, can be reconstructed from tACS-contaminated MEG-recorded data. Most importantly, in both cases the reconstructed activity was virtually identical with the same neural signal when no tACS was applied.

These advances generally allow an investigation of any oscillatory brain response during concurrent tACS. Here, we put our approach to a new test by probing online tACS effects on a special type of rhythmic brain activity known as steady-state responses (SSRs) that are driven by, and thus strictly time-locked, to periodic visual flicker stimulation (Regan, 1989; Norcia et al., 2015).

SSRs have been studied since the early days of EEG research (Adrian and Matthews, 1934). To date, their exact neurophysiological origin is still under debate (Keitel et al., 2014). Whereas some researchers treat SSRs as externally entrained intrinsic neural rhythms, such as the alpha rhythm (Mathewson et al., 2012; Spaak et al., 2014), others suggest that they mainly compose of successive transient sensory evoked responses that add to the ongoing electrophysiological signal (Shah et al., 2004; Capilla et al., 2011). For the purpose of the present study we refrain from endorsing either perspective but simply treat SSRs as stimulus-driven brain oscillations with unique properties outlined below that make them an ideal candidate for a combination with tACS research.

In the spectral domain SSRs can be considered narrow-band responses whose bandwidth can be neglected when considering multiple SSR cycles, i.e., longer stimulation periods. SSRs further comprise a number of (equally narrow-band) higher order harmonics, i.e., spectral components at multiples of the driving frequency that are typically found in frequency-tagging experiments (Appelbaum et al., 2006; Kim et al., 2011; Porcu et al., 2013) and point towards non-linear properties of the visual system (Roberts and Robinson, 2012). A body of research on visual processing has employed SSRs to study, for instance, attentional influences (Müller and Hillyard, 2000; Kim et al., 2007; Störmer and Alvarez, 2014), cognitive load (Jacoby et al., 2012), perceptual segregation (Appelbaum et al., 2006), the aging brain (Quigley and Müller, 2014), inter-stimulus competition (Porcu et al., 2014), as well as object- (Kaspar et al., 2010; Koenig-Robert and VanRullen, 2013), and face processing (Rossion and Boremanse, 2011; Rossion et al., 2012).

In comparison with relatively broadband intrinsic rhythms that are typically targeted in tACS experiments, such as alpha (8–13 Hz), SSRs may be a better fit to the strictly sinusoidally alternating current and the implicit underlying stationarity assumption of cortical oscillations. Online effects of tACS on stimulus-driven oscillatory responses might be more readily observable because the spectral profile of the stimulation, and thus in principle the resulting waveforms, are precisely set by the experimenter.

In the present study we administered tACS while concurrently recording SSRs. To this end, we developed a novel protocol that was optimized to deliver tACS in 2 s intervals concurrently with matched- and different-frequency visual flicker in a classical trial-by-trial experimental paradigm. Based on our previous success in recovering the alpha rhythm during alpha band tACS by means of a beamformer source projection (Neuling et al., 2015), we expected a similar outcome with regard to SSRs during tACS. In line with studies showing alpha power increases after alpha band tACS (Zaehle et al., 2010; Neuling et al., 2013; Vossen et al., 2015), we hypothesized that matching flicker and tACS frequency would lead to pronounced SSR power. The latter hypothesis further entailed the assumption that no effects would be observed when flicker and tACS frequencies did not match.

## Materials and Methods

### Participants

Seventeen healthy participants volunteered for the current study (4 female, mean age 26 years, one left handed). Two had to be excluded due to hardware problems with the stimulation setup, resulting in a final group of 15 analyzed subjects (4 female, 25.5 years, one left handed). The experiment was approved by the local ethics committee of the University of Trento and adhered to the tenets of the Declaration of Helsinki. All participants signed an informed consent prior to the beginning of the experiment.

### Visual Stimuli

**Figure 1:**
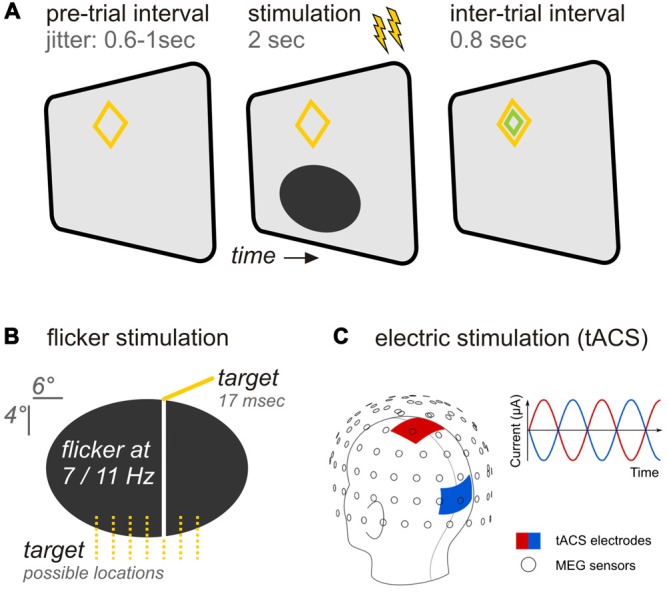
**Experimental design (A) Trial time course.** A pre-trial interval with variable length preceded the stimulation period during which both, flicker and transcranial alternating current (tAC) stimulation (as indicated by flashes) were applied simultaneously for 2 s. Following stimulation an inter-trial interval allowed participants to blink before the next trial started. **(B)** Specifics of the ellipsoid flickering at rates of 7 or 11 Hz. Participants were instructed to report occasional occurrences of vertical bars appearing briefly in one of seven possible locations. **(C)** tACS specifics. The head montage shows the application of tACS delivering electrodes at central (red) and occipital (blue) sites in relation to the magnetoencephalographic (MEG) sensors (adapted from Neuling et al., 2015). In each trial, participants received either no tACS, tACS at the same frequency as the flicker, or tACS at the other frequency.

Participants viewed experimental stimuli back-projected on a translucent screen by a Propixx DLP projector (VPixx technologies, Saint-Bruno, Canada), employing a refresh rate of 120 frames per second and a resolution of 1920 × 1080 pixel (width × height). The stimulation comprised an ellipse (horizontal/vertical diameter = 6.6°/3.3° of visual angle) positioned in the lower visual field at a center-to-center eccentricity of 3° below fixation (Figures 1A,B). A diamond shape (maximum eccentricity = 0.9°) served as a central fixation point. Stimuli were presented against a gray background (RGB: 85, 85, 85).

The ellipse underwent periodic luminance changes (= *flicker*) at rates of either 7 or 11 Hz in the course of each trial: Relative luminance to background oscillated between a minimum of 0% (total black, RGB: 0,0,0) and a maximum of 100% (background gray). Ellipse luminance changed in small increments on each presentation frame to approximate sinusoidal modulations.

We chose our two frequencies within a range that is typically used in frequency-tagging experiments (see Norcia et al., 2015). Both frequencies were hence known to produce SSRs of high signal-to-noise ratios. Further vital to the design of our study was that 7 and 11 Hz SSRs did not produce harmonics that coincided spectrally within the range of frequencies that we analyzed (<50 Hz).

### TACS Parameters

A battery-operated stimulator system connected to rubber stimulation electrodes (DC-Stimulator Plus, NeuroConn GmbH, Ilmenau, Germany) controlled by the stimulation computer was placed outside the magnetically shielded room. It was connected to the stimulation electrodes inside the MEG cabin via the magnetic resonance imaging (MRI) module (NeuroConn). Using the remote input of the stimulator to control the stimulation signal on a trial-by-trial basis, an alternating, sinusoidal current at either 7 or 11 Hz was delivered for 2 s. Stimulation electrodes were centered at electrode positions Cz and Oz of the international 10–20 system (Figure 1C). These positions were chosen for maximal stimulation intensity in the parieto-occipital cortex (Neuling et al., 2012b). The electrodes had a size of 7 by 5 cm and were attached to each participant’s head with a conductive paste (Ten20, D.O. Weaver, Aurora, CO, USA) resulting in impedance values lower than 10 kΩ. The electrode cables were located on the left side of the participant’s head. To keep participants unaware of the electrical stimulation during the experiment, the stimulation intensity was kept below each participant’s sensation and phosphene threshold. To obtain the threshold, the participants were first familiarized with the skin sensation. Afterwards, an intensity of 400 μA (peak-to-peak) was applied at 7 Hz for 30 cycles (4.29 s). Intensity was increased in steps of 100 μA until participants indicated skin sensation or phosphene perception or an intensity of 1500 μA was reached. In the five cases in which the participant already reported a skin sensation at 400 μA, the intensity was reduced to a start level of 100 μA. The staircase procedure resulted in stimulation intensities of *M* 613 *SD* 128 μA. The net tACS stimulation time during the experiment was 10 min 40 s (5′20″ for each stimulation frequency) when summing individual trial stimulation (2 s each).

Note that an inherent difficulty in combining tACS and SSRs lies in the fact that measured effects may depend on the phase relationship of both types of stimulation. Starting electrical and visual stimulation simultaneously and in phase will inadvertently lead to a phase lag in the periodic modulation of neural activity induced by the two methods: whereas electrically induced oscillations will likely have a near-zero phase lag with regard to the driving tACS (Fröhlich and McCormick, 2010; Reato et al., 2010), SSRs will show a substantial phase lag relative to the driving flicker stimulation that depends on the synaptic conduction delays of the visual system from eyes to visual cortex. In the present study, we neglected this tACS-SSR phase lag because: (1) to date, it needs to be shown that an SSR phase lag relative to flicker stimulation can be reliably estimated and remains constant during concurrent tACS; and (2) it went past the scope of our study, namely, demonstrating the feasibility of reconstructing SSRs from MEG recordings contaminated with tACS artifacts at identical frequencies.

### Procedure and Task

We manipulated the factors ellipse *flicker frequency* (7 vs. 11 Hz) and *tACS frequency* (7 vs. 11 Hz) in a fully balanced design. For both flicker frequencies, a sham condition (no-tACS) served as a control condition: while all other parameters remained constant, the stimulator did not receive a signal in the sham tACS trials. Trials of the resulting six conditions were presented in a pseudo-randomized order. In total, each participant ran 480 trials (= 80 trials per condition) divided into eight blocks (~5 min each), separated by self-paced breaks.

During the experiment participants were seated in a comfortable chair and directed gaze towards a screen positioned 1 m in front of them. Experimental trials started with ellipse onset. During the following 2 s the ellipse flickered at a constant rate of either 7 or 11 Hz, dependent on experimental condition. At the end of each trial, a smaller green diamond appeared within the orange fixation diamond for 800 ms indicating participants a favorable time-range to blink before the next trial started (Figure 1A).

Participants were instructed to press a button with the right hand after occasional brief occurrences (16.6 ms/2 frames) of a vertical line superimposed on the ellipse at one of seven pseudo-randomly chosen locations (Figure 1B). Target events appeared in 40% of all trials and, if so, once per trial at a pseudo-randomly chosen time point within an interval starting 500 ms after ellipse onset and ending two frames before stimulus offset. Responses were recorded with an MRI compatible response collector (RESPONSEPixx, VPixx technologies, Saint-Bruno, Canada).

Prior to the main experiment, participants performed at least one prolonged training block (~10 min). After each block, participants received feedback regarding average task performance in terms of hit rate and response speed.

### MEG Data Recording

Electrophysiological data were recorded using a whole head Elekta Neuromag MEG (ElektaOy, Helsinki, Finland) placed in a magnetically shielded room (Vacuumschmelze, Hanau, Germany). Magnetic brain activity was recorded from 102 positions above the head, each comprising a sensor triplet (one magnetometer, two orthogonal planar gradiometers) and sampled at 1000 Hz with an on-line band-pass filter (0.1–330 Hz) active. Before the experiment individual head shapes were acquired for each participant, including fiducials (nasion, left/right pre-auricular point), and around 200 digitized points on the scalp acquired with a Polhemus Fastrak digitizer (Polhemus, VT, USA). During the recording five head position indicator coils (HPIs) tracked the position of the participants’ head.

### MEG Data Analysis

Continuous data were high-pass filtered off-line (Finite Impulse Response (FIR), Kaiser window, cut-off 1 Hz, pass-band 2 Hz) and down-sampled to 512 Hz. Then, epochs of 4 s were cut out, starting 1 s before and ending 3 s after flicker onset. Epochs without tACS stimulation were visually inspected to identify flat or noisy channels as well as epochs containing physiological artifacts (e.g., caused by blinks or muscle activity). Bad channels identified in these trials were excluded from the whole data set.

Because tACS creates a massive electro-magnetic artifact, several orders of magnitude larger than the brain signal (see Neuling et al., 2015), sensor space epochs were projected into source space using linearly constrained minimum variance (LCMV) beamformer filters (Van Veen et al., 1997) before further analyses. To do this, we followed a procedure described here for individual virtual sensors^1^ and extended it to an equally spaced 1.5 cm grid covering the whole brain (see also Neuling et al., 2015, for a similar procedure).

In short, epochs were low-pass filtered at 45 Hz and single epoch covariances estimated and averaged. With the help of the acquired head shapes (see above), individual subjects’ structural magnetic resonance images were aligned to the MEG space, which was subsequently used to create single-shell head models (Nolte, 2003) and lead field matrices. The average covariance, head model, and lead field matrix were used to obtain beamformer filters. This was done separately for each tACS condition—no tACS, 7 Hz tACS, 11 Hz tACS—to optimize the suppression of the artifact. The filters were subsequently multiplied with the individual epochs resulting in source level epochs. We used a 1.5 cm equally spaced grid (889 grid points covering the brain) in Montreal Neurological Institute (MNI) space and warped these positions into individual headspace, which allowed us to average and compute statistics across participants without further interpolation.

### Spectral Analysis

We analyzed SSRs in the frequency domain using two complementary approaches. First, and in accordance with typical SSR analyses (e.g., Appelbaum et al., 2006; Andersen and Müller, 2010), source-level time series were averaged for each participant and condition separately. Fast Fourier Transforms (MATLAB function *fft*) of averaged data within an interval of 0.5–1.5 s relative to SSR onset^2^ yielded complex spectra. Power spectra were obtained by squaring the absolute values of the complex Fourier coefficients. Statistical analyses were performed on SSR amplitudes (square-root of SSR power) divided by the individual mean amplitude across conditions for each frequency. This normalization procedure removed the substantial inter-individual variance in absolute SSR amplitude while retaining the net effects of tACS.

Secondly, we estimated phase locking values (PLV, also referred to as inter-trial phase coherence; Lachaux et al., 1999) for each condition by Fourier-transforming individual epochs first (again selecting data within an interval of 0.5–1.5 s post SSR onset), and then taking the absolute value of the complex mean of the Fourier coefficients for each condition, normalized to unit length:

$$PLV(f)\;=\;\left|\frac{1}{N}\sum_{n\;=\;1}^{N}\frac{c_{n}(f)}{\left|c_{n}(f)\right|}\right|$$

where *c*_n_*(f)* is the complex Fourier coefficient of trial *n* at frequency *f*. Phase locking (= phase synchrony) as a measure of SSR modulation has been introduced to SSR analyses more recently (e.g., Kim et al., 2007). Previous findings indicate differential sensitivities of SSR amplitude and phase synchrony to top-down influences on sensory processing (Kashiwase et al., 2012; Porcu et al., 2013). We thus included SSR phase synchrony to provide a comprehensive description of SSR modulation by concurrent tACS.

In both analyses, we investigated frequencies from 2–50 Hz. The data were zero padded to a length of 8 s to achieve a 0.125 Hz frequency resolution.

### Statistical Analysis

For the behavioral data, responses were considered a “hit” when a button press occurred between 200–1200 ms after target onset. When participants responded in the absence of target presentations responses were classified as false alarms. Behavioral data analyses revealed that participants produced only few false alarms on average (1.3 ± 0.2 per condition). Thus, we based statistical analyses on hit rates (= number of hits divided by total number of targets per condition). Individual hit rates were subjected to a two-way repeated measures analysis of variances (ANOVA) with factors of *flicker frequency* (7 Hz; 11 Hz) and *tACS frequency* relative to flicker frequency (no tACS, same, different).

Reaction times (RTs) of correct responses were analyzed accordingly. Note that RT analyses were based on median RTs per participant and condition to account for the typical left skew of RT distributions.

Spectral source space MEG data (power and PLV) were analyzed with a 3-way repeated-measures ANOVA comprised of the factors *flicker frequency* (7 Hz; 11 Hz), *tACS frequency* relative to flicker frequency (no tACS; same; different) and *SSR harmonic* (fundamental [f]; 2f; 3f; 4f). Amplitudes were normalized per participant and frequency by dividing them by the mean of the three tACS conditions to reduce individual SSR amplitude variability. For all significant main effects and interactions, probabilities were corrected to control for sphericity violations by adjusting the degrees of freedom (Greenhouse and Geisser, 1959). We report original degrees of freedom, corrected *p*-values (*p*_GG_) and the correction coefficient epsilon (ε_GG_). *Post hoc* tests were conducted where appropriate and controlled for multiple comparisons using the false discovery rate (FDR) procedure across all analyses (Benjamini and Hochberg, 1995).

## Results

### Behavioral Measures Independent of tACS Manipulation

Participants detected target events (briefly flashed vertical lines) with comparable hit rates (Table 1) on ellipses flickering at 7 and 11 Hz (main effect *flicker frequency*: *F*_(1,14)_ = 2.14; *p* > 0.05) and different frequencies of simultaneously administered tACS (main effect *tACS frequency*: *F*_(2,28)_ = 1.47, *p*_GG_ > 0.05, ε_GG_ = 0.890). Reaction time analyses revealed a similar pattern: Neither ellipse *flicker frequency* (*F*_(1,14)_ = 0.04; *p* > 0.05) nor *tACS frequency* (*F*_(2,28)_ = 0.49, *p*_GG_ > 0.05, ε_GG_ = 0.822) influenced response speed (Table 1).

**Table 1:** **Average behavioral performance in the visual detection task (*N* = 15)**.

SSR frequency		7 Hz			11 Hz		
tACS frequency*		*No*	*Same*	*Diff*	*No*	*Same*	*Diff*
Hit rate (%)	*M*	68.1	69.4	71.7	66.0	67.1	69.2
	*±SEM*	2.2	3.2	3.2	2.5	3.3	2.4
Reaction time (ms)	*M*	437	428	426	429	432	427
	*±SEM*	14	11	13	10	12	13

M = mean, SEM = standard error of the mean. * Relative to flicker frequency.

In both analyses, interactions of the factors flicker frequency and tACS frequency were insignificant (*F’s* < 1).

### Sensor Level Data Cannot be Analyzed Because of the tACS Artifact

**Figure 2:**
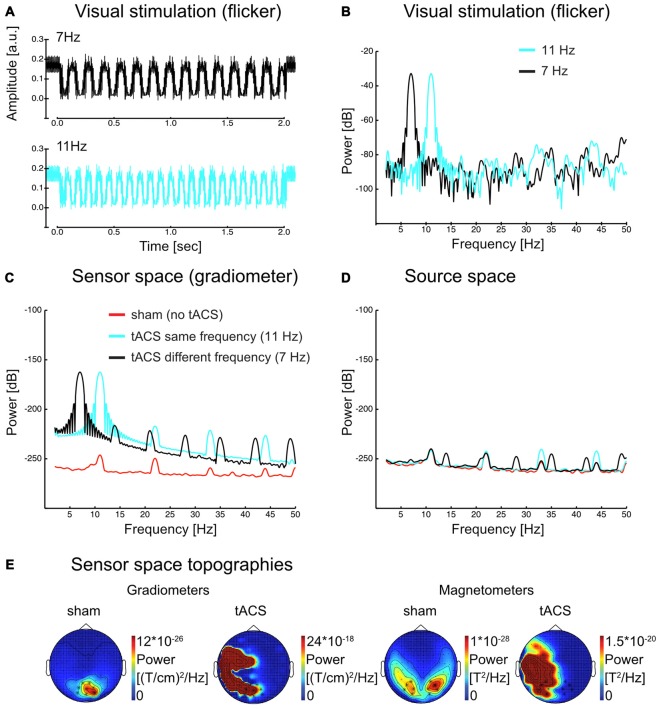
**(A)** Visual flicker as recorded with a photo diode (average over 50 trials in each flicker frequency) and **(B)** power spectrum of the same signal for each flicker frequency. **(C)** Sensor space (gradiometer) MEG data power spectrum in sham and tACS conditions for 11 Hz steady-state response (SSR). Harmonic activity is visible, yet considerably reduced compared to the fundamental. **(D)** Source space data of the same conditions as in **(C)** in visual cortex regions (see Figure 4 for the region of interest). The scale in **(B–D)** is dB (10*log10(power)). **(E)** Sensor space topographies. SSR topographies show occipital activity at the flicker frequency (11 Hz) in the sham condition. During tACS, the artifact is dominating the signal. Note [that] the left lateralized activity is a result of the tACS cables, which were placed on the left side of the participant’s head. Scales change drastically from sham to tACS (factor of around 10^7^–10^8^).

Figures 2A–C illustrate that visual and electrical stimulation signals were dominated by strong fundamental frequency components indicating that both signals were principally sinusoidal (Power spectra in Figure 2 were acquired in a similar manner as for the source space time series, see Materials and Methods section “Spectral analysis”). As Figure 2C demonstrates, the tACS artifact dominated the spectrum at the sensor level and made an analysis of the interaction of SSR and tACS impossible. Source reconstruction by means of LCMV beamforming however suppressed the artifact: in the spectrum in Figure 2D peaks corresponding to the stimulation frequencies were of similar magnitude (compare with Figure 2C). Scalp maps in Figure 2E give an impression of the topographical distribution of the tACS artifact at the fundamental frequency (exemplarily shown here for 11 Hz). Note the massive differences in topography and scale between sham (i.e., SSR only) and tACS conditions. Further note the lateralized topographies during tACS that were caused by currents in the electrode cables fastened to the left side of the participants’ head.

Interestingly, especially in case of tACS the electrical artifact picked up at the sensor level (Figure 2C) also contained higher order harmonic components. These harmonics were several orders of magnitude smaller than the driving frequency component (~60 dB = 40:1). In source-projected data, however, fundamental and harmonic responses were of similar magnitude (Figure 2D). Ultimately, our experiment alone did not allow a further investigation into whether it was the minute stimulation of harmonic components itself or non-linear responses to tACS at the fundamental frequency in the brain that gave rise to neural harmonics (as proposed for SSRs, see Roberts and Robinson, 2012). Considerable tACS harmonics in artifact-removed source reconstructions speak for the latter option, nevertheless. Given the data at hand, in the following, we regard them as genuine brain responses in either case.

### Visual SSRs can be Reconstructed Even with tACS at the Same Frequency

**Figure 3:**
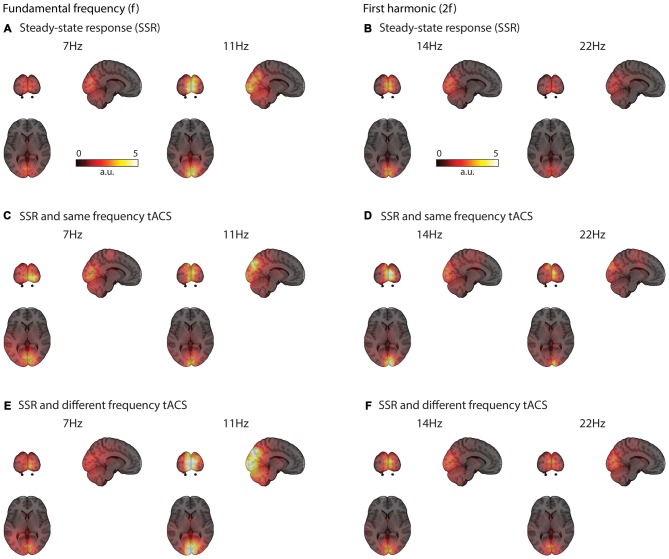
**Grand average functional brain activity (power) evoked by the visual flicker mapped onto a standard montreal neurological institute (MNI) brain.** SSR strongly activates occipital regions **(A)** The activation patterns are similar even with concurrent tACS stimulation at the same **(C)** and different-frequency **(E)** Similar occipital regions are active at the second harmonic (2f) of the SSR **(B,D,F)**.

**Figure 4:**
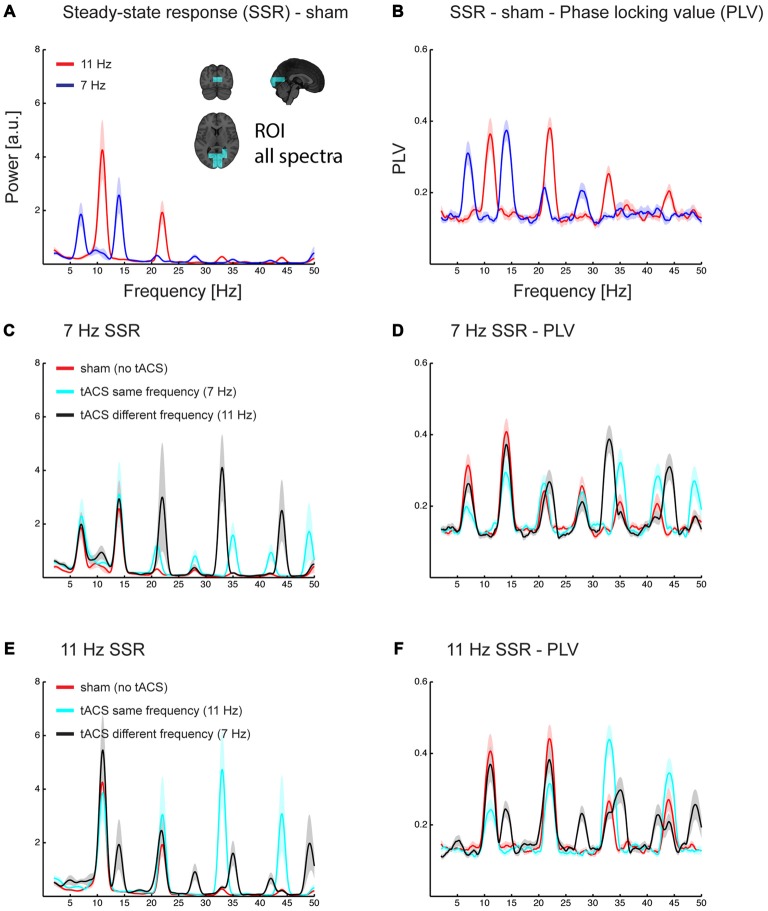
**Grand average spectra of evoked power (A,C,E) and phase locking value (PLV) across trials (B,D,F).** All spectra are the average of estimates in a region of interest covering early sensory visual cortex areas along the Calcarine fissure (see inlet in **A**). Power and PLV are shown for 7/11 Hz SSR without applying tACS **(A,B)** and for all three tACS conditions (no-tACS, same-frequency tACS, and different-frequency tACS) for 7 Hz **(C,D)** and 11 Hz SSR **(E,F)**. Note that tACS seems to differently affect the magnitude for the fundamental and harmonics frequencies. Shaded areas represent standard error of the mean.

**Figure 5:**
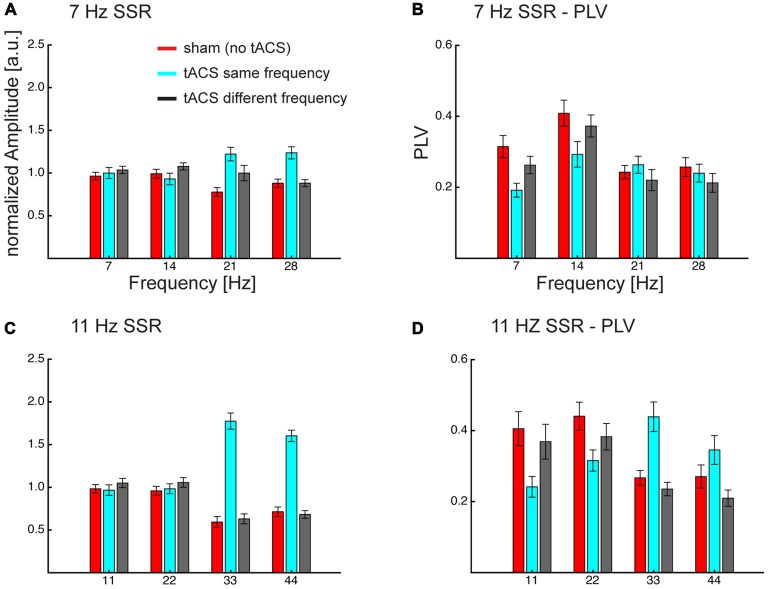
**Grand average normalized amplitudes (A,C) and PLV (B,D) at the visual flicker driven fundamental (*f* = 7/11 Hz) and 3 harmonic (2f, 3f, 4f) frequencies.** Frequency specificity of tACS can be seen in amplitude and PLV, also tACS seems to affect different parts of the flicker driven brain signal. Both measures show that same-frequency tACS is strongly enhancing the harmonics (3f, 4f) and PLV additionally shows a decrease in the lower harmonics. Overall this pattern seems to be more pronounced for 11 Hz SSRs. Note that neither fundamental nor harmonics seem to be affected when the tACS frequency differs from the flicker/SSR frequency. Error bars represent standard error of the mean.

Visual flicker drives brain response at the stimulation frequency and also at harmonics mainly in early visual areas (see Figures 3–5). These responses could be clearly reconstructed with concurrent same- and different-frequency tACS. The neural sources of the SSR were localized to highly comparable regions on the occipital pole (Figure 3).

As mentioned above, spectra of source reconstructed oscillatory activity contained fundamental and harmonic responses elicited by tACS (clearly visible in spectra of conditions in which flicker and tACS frequencies differed; see Figures 4C–F).

### SSR Power and Phase Locking—tACS Affects Fundamental and Harmonic Frequencies Differently

#### SSR Amplitude

An ANOVA, comprised of the factors *flicker frequency* (7 Hz; 11 Hz), *tACS frequency* relative to flicker frequency (no tACS; same; different) and *SSR harmonic* (fundamental [f]; 2f; 3f; 4f) revealed the following effects: a significant main effect of tACS frequency (*F*_(2,28)_ = 26.91, *p*_GG_ < 0.001, ε_GG_ = 0.718), caused by larger amplitudes in the same-frequency tACS condition compared to no- and different-frequency tACS (*p*_FDR_ < 0.05), while there were no significant differences between no and different-frequency tACS. Furthermore, a *flicker frequency × tACS frequency* interaction was significant (*F*_(2, 28)_ = 16.27, *p*_GG_ < 0.001, ε_GG_ = 0.743), explained by the fact that at 7 Hz visual flicker (pooled across harmonics) no-tACS showed smaller amplitudes than same-frequency tACS (*p*_FDR_ < 0.05) but no other significant differences were found, while at 11 Hz both no- and different-frequency tACS were significantly smaller in amplitude than same-frequency tACS (all *p*_FDR_ < 0.05). Furthermore, *an SSR harmonic × tACS frequency* interaction was significant (*F*_(6,84)_ = 36.40, *p*_GG_ < 0.001, ε_GG_ = 0.564) caused by tACS frequency effects at 3f and 4f, with larger amplitudes at same-frequency tACS compared to no- and different-frequency tACS (all *p*_FDR_ < 0.05), while there were no significant tACS frequency effects at the fundamental and 2f (all *p*_FDR_ > 0.05). This pattern was more pronounced with 11 Hz compared to 7 Hz visual flicker (see Figure 5), which resulted in a significant 3-way interaction (*F*_(6,84)_ = 5.35, *p*_GG_ = 0.002, ε_GG_ = 0.604). This was evident in larger differences of no- and different-frequency tACS compared to same-frequency tACS in 3f and 4f (7 vs. 11 Hz, all *p*_FDR_ < 0.05), while contrasts between tACS frequency differences revealed no significant effects at the fundamental and 2f (all *p*_FDR_ > 0.05).

#### SSR Phase Locking

A similar three-way ANOVA on SSR phase locking revealed main effects of *flicker frequency* (*F*_(1,14)_ = 12.73, *p* = 0.003), caused by larger PLVs for 11 Hz compared to 7 Hz, and a main effect of *SSR harmonic* (*F*_(3,42)_ = 16.31, *p*_GG_ < 0.001, ε_GG_ = 0.875), caused by largest PLVs at 2f followed by the fundamental (*p*_FDR_ < 0.05) and 3f (*p*_FDR_ < 0.001) and smallest PLVs at 4f (all *p*_FDR_ < 0.05). Furthermore, the *SSR harmonics × tACS frequency* interaction (*F*_(6,84)_ = 35.70, *p*_GG_ < 0.001, ε_GG_ = 0.562) and the *flicker frequency × tACS frequency* interaction (*F*_(2,28)_ = 7.83, *p*_GG_ = 0.003, ε_GG_ = 0.954) was significant, yet they were further explained by the significant three-way interaction (*F*_(6,84)_ = 5.06, *p*_GG_ = 0.003, ε_GG_ = 0.583). No other effect was significant (all *F* < 2.4, *p* > 0.095).

To resolve the three-way interaction, we conducted two-way ANOVAs on the individual frequencies.

For 7 Hz the ANOVA revealed a significant main effect of *tACS frequency* (*F*_(2,28)_ = 6.89, *p*_GG_ = 0.005, ε_GG_ = 0.888) and of *SSR harmonic* (*F*_(3,42)_ = 14.44, *p*_GG_ < 0.001, ε_GG_ = 0.873). Furthermore, the interaction was significant (*F*_(6,84)_ = 9.13, *p*_GG_ < 0.001, ε_GG_ = 0.742). *Post hoc* tests showed harmonic dependent tACS frequency effects: at the fundamental response the no-tACS condition yielded larger PLVs than same-frequency tACS (*p*_FDR_ < 0.01) and different-frequency tACS (*p*_FDR_ < 0.05) and different-frequency tACS yielded larger PLVs than same-frequency tACS (*p*_FDR_ < 0.01). At 2f no-tACS and different-frequency tACS yielded similar PLVs (*p*_FDR_ > 0.05) but both yielded larger PLVs than same-frequency tACS (*p*_FDR_ < 0.01). At 3f the pattern reversed and showed smaller PLVs for no-tACS compared to same-frequency tACS (*p*_FDR_ < 0.05), but there were no differences between no-tACS and different-frequency tACS and same- and different-frequency tACS (all *p*_FDR_ > 0.05). At 4f there were no differences (all *p*_FDR_ > 0.05).

For 11 Hz the ANOVA revealed a main effect of *SSR harmonic* (*F*_(3,42)_ = 6.39, *p*_GG_ = 0.006, ε_GG_ = 0.623) and a significant *tACS frequency × SSR harmonics* interaction (*F*_(6,84)_ = 27.55, *p*_GG_ < 0.001, ε_GG_ = 0.453). This interaction was caused by a difference in the overall patterns of tACS effects on SSR harmonics: for the fundamental frequency there were no differences between no- and different-frequency tACS (*p*_FDR_ > 0.05) but both showed larger PLVs than the same-frequency tACS condition (all *p*_FDR_ < 0.01). At 2f no-tACS still showed larger PLVs than same-frequency tACS (*p*_FDR_ < 0.05) but no other comparisons were significant. At 3f the pattern observed at the fundamental inversed; although there were no differences between no- and different-frequency tACS (*p*_FDR_ > 0.05), both showed smaller PLVs than the same-frequency tACS condition (all *p*_FDR_ > 0.01). At 4f, no-tACS showed smaller PLVs than same (*p*_FDR_ > 0.01) and larger PLVs than different-frequency tACS (*p*_FDR_ > 0.05), furthermore, same-frequency tACS showed larger PLVs than different-frequency tACS (*p*_FDR_ < 0.01).

### TACS Alters SSR Waveform—An Example

**Figure 6:**
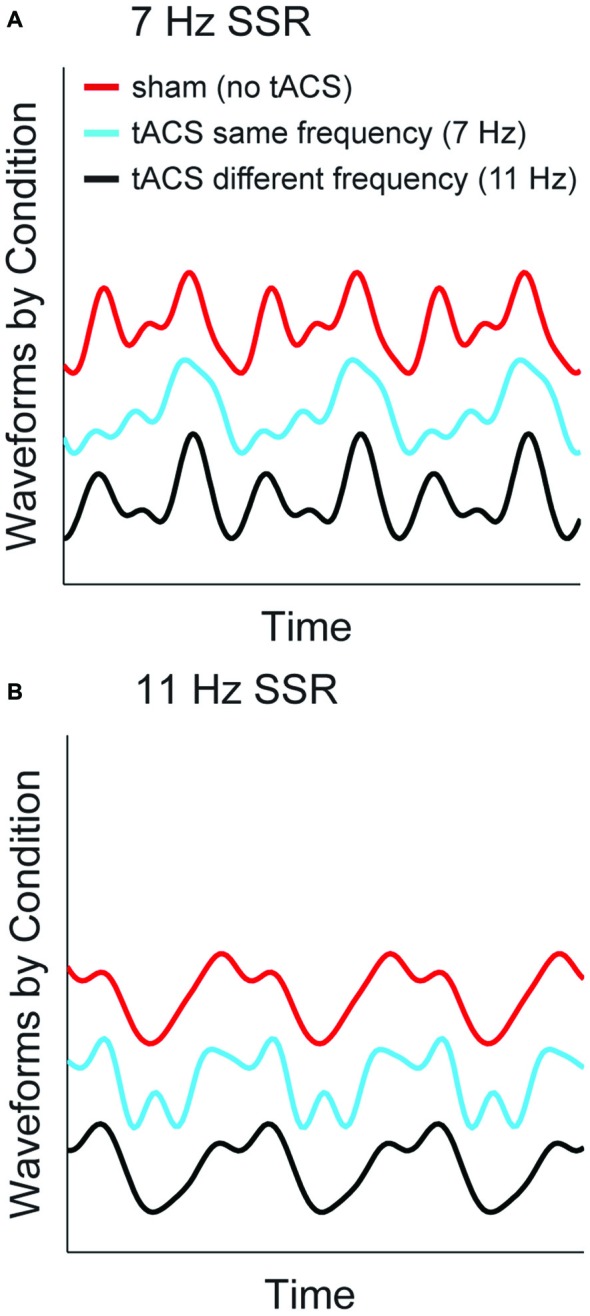
**SSR waveforms reconstructed from spectral components at fundamental and harmonic frequencies.** Graphs show three cycles of the fundamental response of one representative subject for both stimulation frequencies **(A)** 7 Hz SSR **(B)** 11 Hz SSR. Line colors code the three tACS conditions. Note that the shift along the *y*-axis is non-informative and only serves illustrative purposes.

To visualize the specific effects of same-frequency tACS and to illustrate the differential contribution of fundamental and harmonic components to the time domain signal we reconstructed time series waveforms from source-level spectral SSR representations. To this end, we summed the sinusoids described by the (amplitude and phase of) Fourier coefficients at fundamental frequencies and the harmonics up to 4f averaged across voxels in an early visual cortex region of interest (ROI, see Figure 4A). Respective complex Fourier coefficients were derived as described above in the analysis of SSR power (see “Materials and Methods” section “Spectral analysis”). Figure 6 depicts reconstructed waveforms of one representative subject that correspond to three cycles of the respective fundamentals for the three conditions: no-tACS, same-frequency tACS and different-frequency tACS. The no-tACS condition clearly shows the quasi-sinusoidal morphology that gives rise to strong higher order harmonics in spectral decompositions. Whereas no-tACS and different-frequency tACS waveforms show a strong resemblance, same-frequency tACS has a specific influence on SSR morphology.

## Discussion

The present study shows that: (1) recording SSRs in MEG during concurrent tACS, and thus a combination of both methods of brain stimulation, is feasible. To this end, we have implemented a novel tACS protocol that allows intermittent stimulation with frequencies varying in a classical trial-by-trial experimental design; (2) thus recorded SSRs can be reconstructed at the source level by means of LCMV beamforming that effectively removes tACS-introduced artifacts. Importantly, this procedure yields plausible results even when SSR and tACS have identical temporal frequencies; and (3) simultaneous tACS modulates SSRs in a frequency-specific manner: for both stimulation frequencies tested (7 and 11 Hz), same-frequency tACS had the most profound effect on SSRs. The effects of different-frequency tACS instead were largely comparable to a control condition in which no tACS was administered.

In the following we discuss these findings in detail, expose outstanding questions and issues and introduce possible future directions regarding experimental applications.

### Combining Two Rhythmic Stimulation Methods

The first aim of the current study was to provide a proof of principle that brain activity evoked by a rhythmic visual stimulation can be reconstructed with concurrent tACS at the same frequency. Using LCMV beamformers on concurrent MEG-tACS data achieved this aim. As pointed out by Van Veen et al. (1997), the beamformer source reconstruction reduced highly correlated noise thus suppressing the massive tACS sensor artifact (Neuling et al., 2015). Crucially, spatially circumscribed generators of SSRs in early visual cortices remained unaffected (see Figure 3) independently of the applied tACS frequency. This is particularly remarkable in case of matched-frequency tACS because the artifact removal via beamforming could have resulted in a suppression (if not removal) of SSR power itself.

Typically, in electrical stimulation designs the stimulation is applied for a longer period of time (e.g., Zaehle et al., 2010; Neuling et al., 2013; Helfrich et al., 2014b) to yield stable after-effects (but see Vossen et al., 2015). Here, we investigated direct effects of short 2 s tACS trains on brain activity, showing a modulation of the SSR waveform by electrical stimulation (see Figures 4–6). After-effects were out of the scope of the current study. Instead, we aimed to demonstrate immediate and frequency-specific tACS effects following established designs of SSR experiments. Nevertheless, future investigations of on-line effects and additionally registering after-effects and their relationship might help understanding the mechanism of how tACS is modulating brain oscillations and could clarify whether these are based on entrainment or neural plasticity or a combination of both (Vossen et al., 2015).

### tACS Influences a Stimulus Driven Oscillator

Our data suggest a well-circumscribed online modulation of brain oscillations in humans by tACS. Even though in our recent study (Neuling et al., 2015) we showed that endogenous oscillations and their modulations can be recovered during same frequency tACS, modulations of brain activity caused by tACS were not in our focus.

Many studies showed behavioral consequences or electrophysiological changes in other frequencies (Helfrich et al., 2014a; Voss et al., 2014), but a neurophysiological proof for the stimulation frequency is still missing. Note that Helfrich et al. (2014b) did not include a control condition, and thus the 10 Hz increase during stimulation might still be a result of the stimulation artifact itself. This fact is underlined by work from the same group (Helfrich et al., 2014a) in which signals around the stimulation frequency had to be notch-filtered. Here, however, we stimulated at different but spectrally close frequencies (7, 11 Hz) and used a fully balanced design. Thus any artifactual effects caused by the tACS would have been evident in the analysis when SSR and tACS were presented at different frequencies, an artifact which would have additionally spread across the spectrum (see also Figure 2). However, we did not observe such an artifact and effects of tACS were limited to matched frequency stimulations.

More specifically, we found that matched frequency stimulation reduced phase synchrony of fundamental (1f) and second harmonic (2f) SSR components while boosting evoked power and phase synchrony of third and fourth harmonic components. This result contrasts with our initial hypotheses of tACS-induced power increases of fundamental SSR components. In the following we suggest that our finding critically depends on the phase relationship between tACS and SSR.

Studies targeting the alpha rhythm with tACS are based on the assumption that the generative neural process underlying alpha will align its phase with the external electrical pacemaker (Fröhlich and McCormick, 2010; Neuling et al., 2012a, 2013). A similar phase alignment for SSRs is unlikely because SSR phase is itself strictly locked to the driving visual stimulation. Therefore, the phase of concurrent matching frequency tACS and SSR phase can differ in principle. For the present study we assumed a fixed phase relationship between tACS and SSRs for both stimulation frequencies and across participants. However, SSRs have been shown to require a number of cycles to fully build up (i.e., reach maximum amplitude e.g., Regan, 1989) whereas the flow of electrical currents introduced by tACS is assumed to have instantaneous effects (Fröhlich and McCormick, 2010; Reato et al., 2010). Due to inter-individual neuro-anatomical differences (e.g., conduction delays in early visual processing pathways) SSR phase might jitter between participants. Although we have taken into account the build-up time by analyzing data epochs only during which the SSR was fully established we cannot exclude the possibility that SSR and tACS phase differed substantially and with a variable lag between participants.

In fact, a considerable tACS-SSR phase lag is a possible explanation for our finding of reduced phase locking in 1f and 2f SSR components and enhanced contributions of higher order harmonics during matched frequency stimulation. The two stimulation techniques forced entrainment (here phasic alignment) in similar areas but at different times. Put differently, neural activity evoked by a visual stimulus will peak shortly after maximal tACS (i.e., current peak or trough). As slight timing differences will be considerable parts of the SSR cycle this possibly affects the lower harmonics more strongly. The extent to which the SSR alignment is impaired by tACS probably varies from trial to trial, which consequentially leads to lower phase locking. In turn, boosted higher harmonics could be explained by tACS induced distortions of the SSR waveforms towards less sinusoidal morphologies (see Figure 6).

### Open Questions and Future Directions

Thus far only human experimental study evidence has been provided using the LCMV beamformer approach with tACS (Neuling et al., 2015) and modeling and phantom measurements only exist for synthetic-aperture magnetometry beamformers (Soekadar et al., 2013). To know exactly how well the LCMV beamformer performs (i.e., reducing the tACS artifact and reconstructing the true source) phantom measurements are essential and more methodological studies need to be performed.

Above we laid out consequences of a possible tACS-SSR phase lag in our study. Future studies should thus implement methods to first estimate individual SSR phase and then re-align flicker stimulation with tACS as to minimize and standardize the phase lag. Furthermore, one could systematically vary the phase lag to test, for instance, whether out-of-phase stimulation produces cancellation effects.

Another aspect of the present study was that our tACS mainly targeted SSR components at fundamental frequencies (with only weak tACS at harmonic frequencies, cf. Figure 2) although visual stimulation also led to pronounced oscillatory components at harmonic frequencies. Harmonic components are typically found in SSR recordings (Appelbaum et al., 2006; Kim et al., 2011; Porcu et al., 2013) and may have their origin in non-linearities of the visual system (Roberts and Robinson, 2012; Norcia et al., 2015). Considering the fact that our results show a complex relationship of matched-frequency tACS with all corresponding SSR components it might be worthwhile to target specific harmonics driven by the stimulus. Conversely, one could also take into account the harmonic composition of SSRs and use a tACS signal that matches the spectral profile, i.e., consists of a superposition of sines that optimally resembles the SSR waveform.

Here, a detection task was simply employed to keep the subjects’ attention on the visual stimuli. The targets were distributed randomly across tACS and SSR phase, thus no behavioral effects were expected. Yet, many studies showed behavioral consequences of tACS phase on perception (Neuling et al., 2012a; Riecke et al., 2015). Recently, similar effects have been presented using visual (Mathewson et al., 2012; Spaak et al., 2014) and also auditory stimuli (Henry and Obleser, 2012; Henry et al., 2014). Basically, detection performance of a low contrast targets depended on the phase of a rhythmic stimulus in which the targets are embedded. A combination of both lines of research may provide evidence as to whether sensory and tACS entrainment work in a similar manner; whether they can be interactive and even increase the, typically small, behavioral effects. To conduct these studies, however, it will be vital to reliably estimate the stimulus-to-brain phase lag.

## Conclusion

Our study demonstrated that reconstructing visual SSRs from MEG recordings during simultaneously administered tACS is possible, even when both match in temporal frequency. tACS influenced SSRs mainly by reducing phase synchrony for the fundamental and second harmonic. At the same time higher order harmonic responses were increased in power and phase synchrony. Importantly, the present results provide further evidence for online effects of tACS on human mass-neuronal rhythmic activity. They open new avenues in studying perception and cognitive influences thereof through causal interference with stimulus-entrained brain rhythms.

## Author Contributions

PR, CK, CL, NW, and TN conceived the experiment. PR, CL, and TN performed the research. PR and CK analyzed the data. All authors interpreted the data and wrote the article. All authors approved the final version of the manuscript.

## Conflict of Interest Statement

The authors declare that the research was conducted in the absence of any commercial or financial relationships that could be construed as a potential conflict of interest.

## Footnotes

^1^http://www.fieldtriptoolbox.org/tutorial/shared/virtual_sensors

^2^The steady-state response has a certain build-up time and is initially overlapped by the event-related response. To get an estimate of the “true” steady-state the initial part of the epoch is typically ignored (e.g., Regan, 1989; Kim et al., 2011; Keitel et al., 2013). Similar reasoning holds for the late part of the epoch where offset responses can contaminate the frequency estimate.
